# Association between mammillary body atrophy and memory impairment in retired athletes with a history of repetitive mild traumatic brain injury

**DOI:** 10.1038/s41598-024-57383-6

**Published:** 2024-03-26

**Authors:** Mari Miyata, Keisuke Takahata, Yasunori Sano, Yasuharu Yamamoto, Shin Kurose, Manabu Kubota, Hironobu Endo, Kiwamu Matsuoka, Kenji Tagai, Masaki Oya, Kosei Hirata, Fumie Saito, Masaru Mimura, Koji Kamagata, Shigeki Aoki, Makoto Higuchi

**Affiliations:** 1 Department of Functional Brain Imaging, Institute for Quantum Medical Science, Quantum Life and Medical Science Directorate, National Institutes for Quantum Science and Technology (QST), Chiba, Japan; 2 Department of Radiology, Juntendo University School of Medicine, Tokyo, Japan; 3 Department of Psychiatry, Kyoto University Graduate School of Medicine, Kyoto, Japan; 4 Department of Neuropsychiatry, Keio University School of Medicine, Tokyo, Japan

**Keywords:** Cognitive ageing, Cognitive neuroscience, Diseases of the nervous system, Learning and memory

## Abstract

Cognitive dysfunction, especially memory impairment, is a typical clinical feature of long-term symptoms caused by repetitive mild traumatic brain injury (rmTBI). The current study aims to investigate the relationship between regional brain atrophy and cognitive impairments in retired athletes with a long history of rmTBI. Overall, 27 retired athletes with a history of rmTBI (18 boxers, 3 kickboxers, 2 wrestlers, and 4 others; rmTBI group) and 23 age/sex-matched healthy participants (control group) were enrolled. MPRAGE on 3 T MRI was acquired and segmented. The TBV and TBV–adjusted regional brain volumes were compared between groups, and the relationship between the neuropsychological test scores and the regional brain volumes were evaluated. Total brain volume (TBV) and regional brain volumes of the mammillary bodies (MBs), hippocampi, amygdalae, thalami, caudate nuclei, and corpus callosum (CC) were estimated using the SPM12 and ITK–SNAP tools. In the rmTBI group, the regional brain volume/TBV ratio (rmTBI vs. control group, Mann–Whitney U test, p < 0.05) underwent partial correlation analysis, adjusting for age and sex, to assess its connection with neuropsychological test results. Compared with the control group, the rmTBI group showed significantly lower the MBs volume/TBV ratio (0.13 ± 0.05 vs. 0.19 ± 0.03 × 10^−3^, p < 0.001). The MBs volume/TBV ratio correlated with visual memory, as assessed, respectively, by the Rey–Osterrieth Complex Figure test delayed recall (ρ = 0.62, p < 0.001). In conclusion, retired athletes with rmTBI have MB atrophy, potentially contributing to memory impairment linked to the Papez circuit disconnection.

## Introduction

Contact-sport athletes who receive repetitive concussive and subconcussive blows to the brain can develop cognitive and behavioral impairments after retirement; these were formerly described as punch–drunk syndrome or dementia pugilistica^1,2^. Repetitive mild traumatic brain injury (rmTBI) differs from single moderate or severe TBI in its clinical characteristics from acute to chronic phase, the pattern of recovery, and long-term sequelae, and is a risk factor for late-onset and long-lasting neurocognitive disturbances^3^ that often persist throughout life. Among the diverse cognitive impairments that follow rmTBI, the most frequently observed symptom is memory impairment, which leads to a reduced quality of life for retired athletes^2^. However, the underlying mechanisms linking rmTBI exposure through contact sports to persistent memory impairment is poorly understood (e.g., white matter dysfunction, inflammation, aging effects, and neurodegenerative changes)^4^

Previous neuroimaging modalities, such as computed tomography (CT) and conventional magnetic resonance imaging (MRI), have shown ventricular enlargement, cavum septum pellucidum, and cavum vergae^5,6^, which have been suggested to be signs indicating past exposure to rmTBI. However, the sensitivity of sports-related brain imaging findings is not sufficient for diagnosis due to a large amount of diversity in the neuropathological changes caused by rmTBI and the interindividual variability in severity^7^. In addition, in some contact sports, there are no substantial structural abnormalities on MRI^8,9^. Therefore, it may be suggested that consistent findings related to rmTBI are not fully available. Furthermore, there is no clear relationship between these findings and cognitive testing scores^10,11^. These problems make the diagnosis of long-term cognitive dysfunction due to rmTBI extremely difficult.

A typical delayed-onset condition caused by rmTBI is chronic traumatic encephalopathy (CTE). McKee et al. reported that postmortem CTE brains manifest broad and various macroscopic features, including ventricular enlargement, cavum septum pellucidum or cavum vergae, frontal and temporal atrophy, thinning of the hypothalamic floor and corpus callosum (CC), and shrinkage of mammillary bodies (MBs)^12,13^. The MBs transmit information from the hippocampus to the anterior thalamic nuclei through the mammillothalamic tract. The CC is anatomically located between the fornix and the cingulate gyrus. These are vital links in the memory system within the Papez circuit^14^. Prior concussion is a risk factor for increased hippocampal atrophy and the development of mild cognitive impairment in former National Football League athletes, with an average age in their late 50 s^15^. However, there have been no reports on whether memory impairment in retired athletes with rmTBI is associated with atrophy of brain regions comprising the Papez circuit such as MBs and CC.

The purpose of this study was to identify changes in the regional brain volume related to long-term exposure to rmTBI in retired athletes. This study also evaluated whether changes in regional brain volumes are associated with neurocognitive dysfunction.

## Methods

### Approval

This study was approved by the Institutional Review Board of the National Institute of Radiological Sciences, Chiba, Japan. The study was registered with the University Hospital Medical Information Network Clinical Trials Registry (UMIN 000013517 and UMIN 000030248). All research was performed in accordance with relevant guidelines/ regulations and the Declaration of Helsinki. Written informed consent was obtained from each participant after providing a complete explanation of the study.

### Participants

Participants with rmTBI were recruited from hospitals affiliated to the Keio University School of Medicine and other general hospitals in the metropolitan Tokyo area. Those enrolled in this study fulfilled the following criteria^4^: (i) had a history of head injury, engaged in contact sports for > 2 years, and received multiple concussions; (ii) age ≥ 20 years; (iii) absence of any neuropsychiatric or neurological disorders before or within 1 year of the head injury; and (iv) absence of severe physical diseases. The exclusion criteria included a history of other neurological diseases, an inability to obtain adequate images due to artifacts, and MRI abnormalities. Thus, we excluded one participant with rmTBI due to inadequate image quality. None of the rmTBI participants displayed chronic trauma-related lesions (e.g., cerebral contusions, traumatic subdural hematoma, or microbleeds) on conventional T2-weighted imaging (T2WI), fluid-attenuated inversion recovery (FLAIR), or susceptibility-weighted imaging (SWI), and no participants were excluded due to MRI abnormalities. Age- and sex-matched healthy control participants without a history of TBI or other neurological/psychiatric diseases were recruited from the volunteer association of the National Institutes for Quantum Science and Technology. All healthy control (HC) participants had normal results on conventional imaging.

### Image acquisition

All studies were performed using a 3 T MRI system (MAGNETOM Verio 3 T, Siemens Healthcare; Erlangen, Germany) equipped with a 32-channel head coil. T1-weighted three-dimensional (3D) magnetization-prepared rapid acquisition gradient echo (MPRAGE) scans were acquired using the following parameters: field of view = 250 mm, TR/TE = 2300/2 ms, 512 × 512 matrix, slice thickness = 1 mm, acquisition time = 4 min 33 s.

### Quantitative volumetric analysis

**Figure 1:**
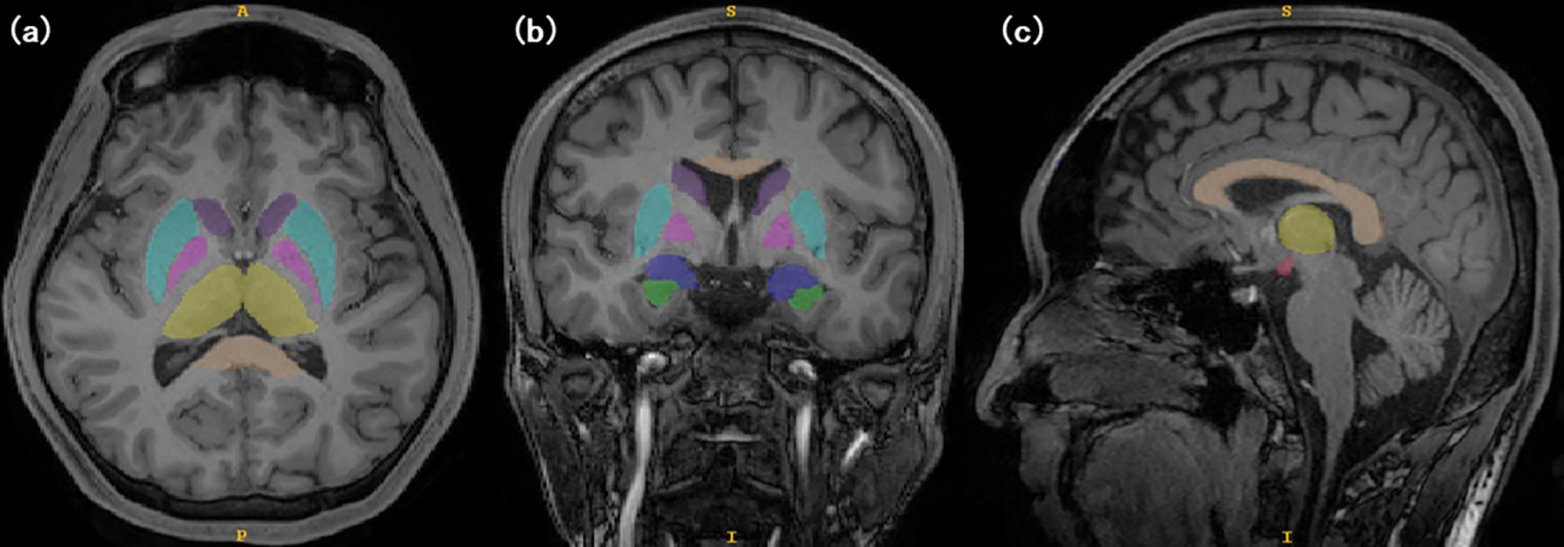
Segmentations in ITK–SNAP show the same (**a**) axial, (**b**) coronal, and (**c**) sagittal slice for a single-term participant (purple: caudate nuclei, light blue: putamina, pink: globus pallida, yellow: thalami, orange: corpus callosum; blue: amygdalae; green: hippocampi; red: mammillary bodies).

Two readers (Reader 1: 17, Reader 2: 13 years of experience in diagnostic neuroradiology) blinded to the participants’ information conducted manual 3D segmentation of the MPRAGE using ITK–SNAP (Insight segmentation and registration toolkit–Snake automatic partitioning; Penn Image Computing and Science Laboratory, University of Pennsylvania, Philadelphia, PA)^16^. The DICOM datasets were imported into the ITK–SNAP and presented in sagittal, coronal, and axial slices. The open-source medical imaging program ITK–SNAP is based on geodesic active-contour and region competition methods; it provides manual and semi-automatic tools to measure the volumes of anatomical regions of interest^16^. The program has been validated previously for volumetric and morphometric analyses of the caudate nucleus^17^. The programs computed the volume of the lesions in cubic millimeters. Two readers independently measured the following volumes as brain structures reported abnormalities in the CTE pathology^13^: the MBs, caudate nucleus, putamina, globus pallidus, talus, hippocampus, amygdala, and CC (Fig. 1). Total brain volume (TBV) was estimated using SPM12 (Wellcome Department of Cognitive Neurology, Institute of Neurology; London, United Kingdom), which is available online at https://www.fil.ion.ucl.ac.uk/spm/).

### Cognitive assessments

The cognitive function of the rmTBI group was assessed using the following neuropsychological tests described in our previous report^4^: the Mini-Mental State Examination for global cognitive function; the logical memory in the Wechsler Memory Scale–revised test (LM; immediate and delayed recall) for verbal memory; the Rey–Osterrieth Complex Figure Test (ROCFT; copy and delayed recall) for visual memory; the digit span (forward and backward) for attentional function; and the verbal fluency test (category and initial letter), Wisconsin Card Sorting Test (WCST, Keio Version), and Stroop Test (I, II, and III) for executive function. Data from the Stroop Test were missing for one participant.

### Statistical analysis

The characteristics of the participants are presented as the mean ± standard deviation (range) for each item. The Mann–Whitney U test was used to compare the TBV, regional brain volumes, and ratio of the regional brain volume to the TBV (the regional brain volume/TBV ratio) between the rmTBI and HC groups. In the rmTBI group, the regional brain volume/TBV ratio, which was p < 0.05 by the Mann–Whitney U test, was subjected to partial correlation analysis using age and sex as covariates to evaluate its relationship with cognitive assessment test results, and sex as covariate with other clinical parameters. The Bonferroni-corrected significance was set at p < 0.006 for comparison analysis and p < 0.005 for correlation analysis to account for multiple testing, and a lenient threshold p < 0.05 was considered to suggest a causal relationship. Interobserver repeatability between the two readers for quantitative assessment was evaluated using the intraclass correlation coefficient (ICCs) calculation mode l—a single rater, absolute agreement, two-way random-effects model. Values less than 0.5 indicate poor reliability, values between 0.5 and 0.75 suggest moderate reliability, values between 0.75 and 0.9 indicate good reliability, and values greater than 0.90 indicate excellent reliability^18^. All statistical analyses were performed using the IBM Statistical Package for Social Sciences (SPSS) Statistics (version 29) software (SPSS Inc., Chicago, IL, USA).

## Results

There were 27 rmTBI participants, with a mean age of 43.9 ± 10.9 (31–69) years and 13.7 ± 2.4 (9–18) years of education; of these, 24 (88.9%) were male. Their sports professions were boxing (n = 18, 66.7%), kickboxing (n = 3, 11.1%), wrestling (n = 2, 7.4%), snowmobiling (n = 1, 3.7%), rugby (n = 1, 3.7%), ice hockey (n = 1, 3.7%), and American football (n = 1, 3.7%). The mean duration since the first injury was 24.0 ± 9.5 (9–47) years, and duration of exposure to rmTBI 15.4 ± 9.4 (12–25) years. No rmTBI participants had a history of severe and moderate TBI, family history of hereditary neurodegenerative disease. Three of rmTBI particiants were diagnosed with an alcohol use disorder, but none had a history of other substance use disorders. The HC group included 23 participants with a mean age of 45.1 ± 14.0 (23–69) years; of these, 20 (87.0%) were male. The data for the characteristics of the rmTBI and control participants are summarized in Table 1.

**Table 1:** Participants characteristics.

	Retired athletes with rmTBI	HC
Participants, n	27	23
Sex, male, n (%)	24 (88.9%)	20 (87.0%)
Age, y, Mean ± sd, range	43.9 ± 10.9, 31–69	45.1 ± 14.0, 23–69
Year of education, y, mean ± SD, range	13.7 ± 2.4, 9–18	ｰ
Sports professions, n	Boxing 18, Kickboxing 3, Wrestling 2, Snowmobiling 1, Rugby 1, Ice hockey 1, American football 1	ｰ
Years since the first injury, y, mean ± SD, range	26.4 ± 11.6, 9–47	ｰ
Duration of exposure to rmTBI, y, mean ± SD, range	15.4 ± 9.4, 12–25	ｰ
Past history of neuropsychiatric diseases	Alcohol use disorder 3 (11.1%)	ｰ
MRI findings on T2WI/FLAIR; DWMH (Fazekas grade 0/1/2/3)	17/7/3/0	15/6/2/0

*rmTBI* repetitive mild traumatic brain injury, *HC* healthy control, *y* year(s), *SD* standard deviation, *T2WI* T2 weighted image, *FLAIR* fluid attenuated inversion recovery, *DWMH* deep white matter hyperintensities.

**Figure 2:**
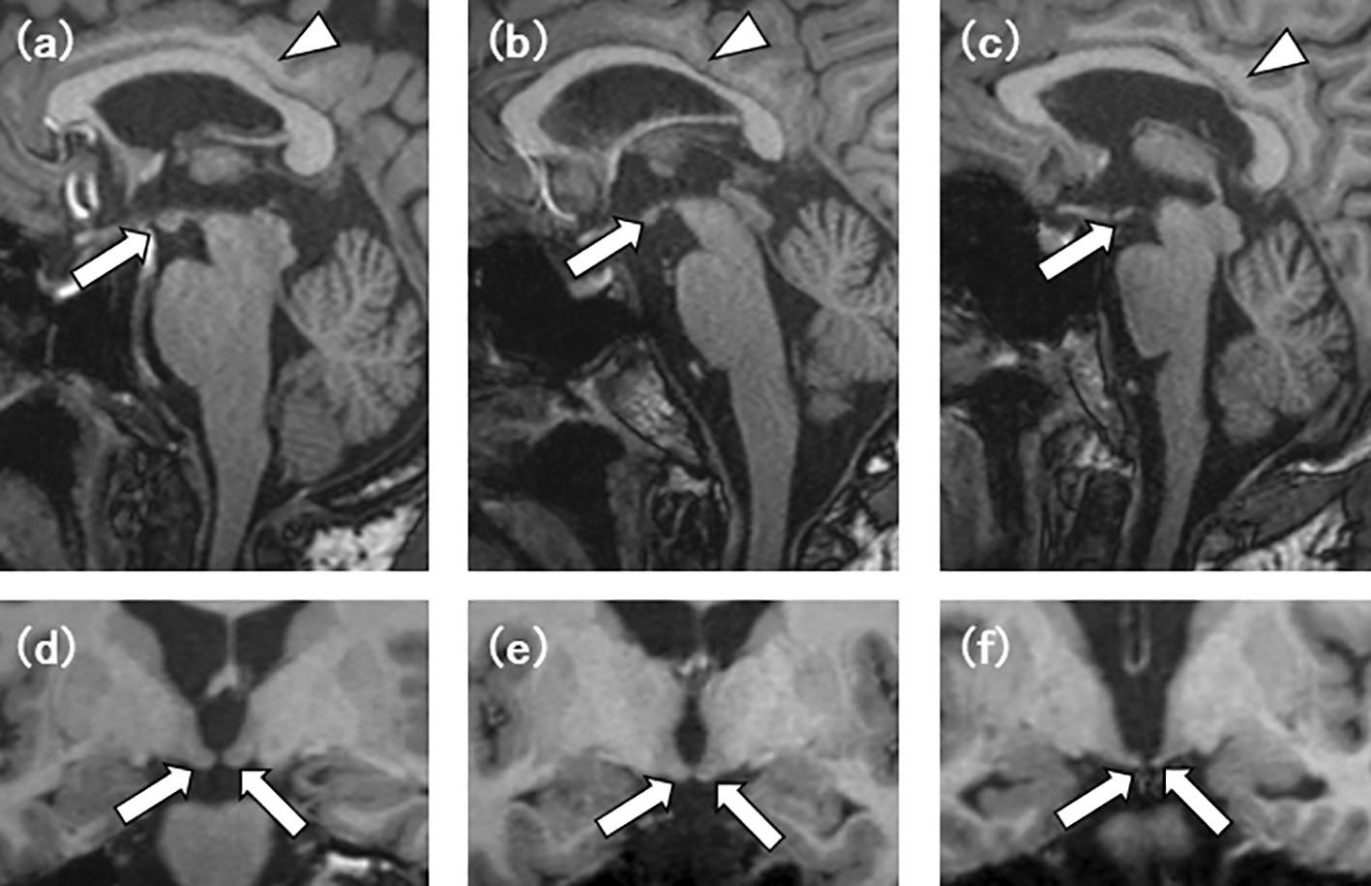
Comparison of the mammillary bodies (MBs) and the corpus callosum (CC) on sagittal (**a**–**c**) and coronal (**d**–**f**) images. (**a**,**d**) A control participant in their 40 s. (**b**,**e**) A professional kickboxer in their 40 s with mild atrophy of the MBs and the CC. (**c**,**f**) A professional boxer in their 40 s with severe atrophy of the MBs and the CC.

Table 2 shows the comparison results between the rmTBI and HC groups. There was no significant between-group difference in TBV (rmTBI vs. HC: 1.12 ± 0.13 vs. 1.17 ± 0.12 × 10^3^ cm^3^). However, the regional brain volumes of the MBs and CC were significantly smaller in the rmTBI group than in the HC group (rmTBI vs. HC: 0.14 ± 0.05 vs. 0.22 ± 0.03 cm3 for the MBs and 13.9 ± 1.77 vs. 15.9 ± 2.32 cm^3^ for CC; p < 0.001), and that of the thalami were causally smaller in the rmTBI group, as assessed by Reader 1 (13.6 ± 2.08 vs. 14.9 ± 2.01 cm^3^; p = 0.04, not significant after Bonferroni correction) (Fig. 2). The other regional brain volumes showed no significant difference. The ratio of the MBs /TBV was significantly smaller in the rmTBI group than that in the HC group (0.13 ± 0.05 vs. 0.19 ± 0.03 × 10^–3^; p < 0.001), and the CC/TBV was smaller in the rmTBI group, as assessed by Reader 1 (12.5 ± 1.63 vs. 13.6 ± 1.81 × 10^–3^; p = 0.03). The other regional volume-to-TBV ratios showed no significant difference. See the result of Reader 2 to the Supplemental Table 1.

**Table 2:** Group differences in brain volumes compared with retired athletes with rmTBI and HC in Reader 1.

	Volume, cm^3^, mean ± SD			Regional volume/TBV ratio, × 10^−3^ mean ± SD		
	Retied athletes with rmTBI	HC	p value	Retied athletes with rm TBI	HC	p value
	n = 27	n = 23		n = 27	n = 23	
TBV	1.12 ± 0.13 × 10^3^	1.17 ± 0.12 × 10^3^	ns	–	–	–
MB	0.14 ± 0.05	0.22 ± 0.03	< 0.001**	0.13 ± 0.05	0.19 ± 0.03	< 0.001**
Put	11.0 ± 1.55	11.2 ± 1.24	ns	9.85 ± 1.11	9.61 ± 0.86	ns
GP	3.18 ± 0.43	3.14 ± 0.48	ns	2.87 ± 0.44	2.70 ± 0.44	ns
CN	8.65 ± 1.37	9.30 ± 1.14	ns	7.74 ± 0.79	7.94 ± 0.73	ns
Thal	13.6 ± 2.08	14.9 ± 2.01	0.04*	12.2 ± 1.22	12.7 ± 1.25	ns
Hipp	7.41 ± 1.05	7.73 ± 1.10	ns	6.65 ± 0.68	6.61 ± 0.77	ns
Amy	3.89 ± 0.66	4.01 ± 0.55	ns	3.48 ± 0.45	3.44 ± 0.46	ns
CC	13.9 ± 1.77	15.9 ± 2.32	< 0.001**	12.5 ± 1.63	13.6 ± 1.81	0.03*

*rmTBI* repetitive mild traumatic brain injury, *HC* healthy control, *SD* standard deviation, *TBV*, total brain volume, *ns*, not significant, *MB* mammillary bodies, *Put* putamina, *GP* globus pallida, *CN* caudate nuclei, *Thal* thalami, *Hipp* hippocampi, *Amy* amygdalae, *CC* corpus callosum **p < 0.006, *p < 0.05.

**Figure 3:**
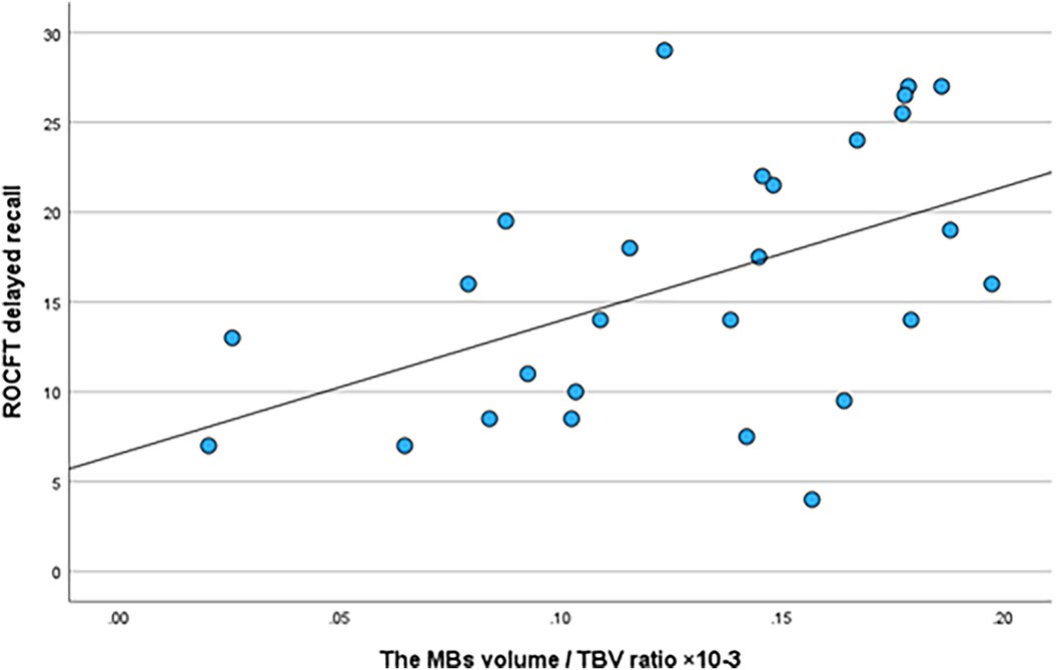
The correlation between the ratio of the mammillary bodies volume to the total brain volume (the MBs/TBV ratio) and the Rey–Osterrieth complex figure test (ROCFT) delayed recall.

The cognitive scores of the rmTBI group are shown in Table 3. In partial correlation analysis, the ratio of the MBs volume/TBV was positively correlated with the results of the ROCFT delayed recall (r = 0.62, p < 0.001) (Fig. 3), and was a trend of correlation with LM for delayed recall (r = 0.51; p = 0.01, not significant after Bonferroni correction); it was not correlated with clinical parameters or other cognitive assessment results. The CC volume/TBV ratio was not correlated with any clinical data or any cognitive assessment results (p > 0.05).

**Table 3:** Correlation analysis of brain volumes and clinical parameters in retired athletes with rmTBI in Reader 1.

	Mean ± SD, range	the MB volume/TBV ratio		the CC volume/TBV ratio	
		r	p value	r	p value
Age	43.9 ± 10.9, 31–69	0.15	ns	0.31	ns
Year of education	13.7 ± 2.4, 9–18	− 0.03	ns	0.12	ns
Years since the first injury	26.4 ± 11.6, 9–47	0.11	ns	0.24	ns
Duration of exposure to rmTBI	15.4 ± 9.4, 12–25	0.27	ns	− 0.03	ns
Cognitive assessments, score					
MMSE	26.5 ± 3.1, 15–30	0.37	ns	0.24	ns
LM					
Immediate recall	8.8 ± 5.0, 0–20	0.37	ns	0.13	ns
Delayed recall	6.2 ± 5.4, 0–20	0.51	0.010*	0.18	ns
ROCF test					
Copy condition	34.8 ± 1.9, 29–36	0.06	ns	0.03	ns
Delayed recall	16.1 ± 7.3, 4–29	0.62	< 0.001**	− 0.06	ns
WCST					
Categories achieved	4.1 ± 2.2, 0–7	0.33	ns	− 0.11	ns
Stroop					
I	16.1 ± 4.0, 10–22	− 0.07	ns	0.11	ns
II	21.0 ± 7.0, 10–35	− 0.04	ns	0.10	ns
III	27.5 ± 15.6, 15–87	− 0.03	ns	0.01	ns

*rmTBI* repetitive mild traumatic brain injury, *MB* mammillary bodies, *TBV* total brain volume, *CC* corpus callosum, *SD* standard deviation, *ns* not significant, *MMSE* mini-mental state examination, *LM* logical memory, *ROCF* test, Ray–Osterrieth complex figure test, *WCST* Wisconsin card sorting test Keio version **p < 0.005, *p < 0.05.

The ICCs and 95% confidence intervals for the regions were MBs: 0.96 (0.93–0.97), putamina: 0.94 (0.80–0.97), globus pallida: 0.86 (0.79–0.90), caudate nuclei: 0.96 (0.95–0.98), thalami: 0.97 (0.97–0.99), hippocampi: 0.96 (0.94–0.97), amygdalae: 0.94 (0.91–0.96), and CC: 0.96 (0.92–0.98). The interobserver reproducibility between the two reviewers for measurements demonstrated good agreement for the globus pallida and excellent agreement for the other evaluated brain regions. See the result of Reader 2 to the Supplemental Table 2.

## Discussion

This study evaluated regional brain volumes in retired athletes with a history of rmTBI and found that TBV-adjusted brain volumes of the MBs and CC were lower than those in HCs. In addition, the TBV-adjusted MB volume correlated with scores of the ROCFT delayed recall, and the TBV-adjusted CC volume did not correlate with any cognitive assessment results in rmTBI participants. Furthermore, the good or excellent interobserver agreement suggested that MRI-based evaluation methods for regional brain volumes using ITK–SNAP are applicable for individualized assessment of rmTBI. To the best of our knowledge, this is the first report to demonstrate MB atrophy and its association with the severity of memory impairment on an individual basis in rmTBI participants.

The MBs form a vital link in a putative memory system, comprising projections from the hippocampus to the MBs, connecting to the anterior thalamic nuclei^14^ through the mammillothalamic tract. Recent studies describing the role of the MBs in memory emphasize the importance of the hippocampal inputs to the region; the MBs are often referred to as part of an “extended hippocampal system”^14^. The presence of MB atrophy has been reported in various conditions, including Korsakoff’s syndrome^19^, postresection of colloid cysts in the third ventricle^20^, Alzheimer’s disease (AD)^21^, schizophrenia^22^, heart failure^23^, and sleep apnea^24^. Furthermore, recent evidence suggests that the MBs are important for memory highlights the memory problems associated with these conditions and the need to accommodate such problems^25^.

CTE is a persistent condition caused by rmTBI. The gross pathological findings of CTE include ventricular enlargement, cavum septum pellucidum or cavum vergae, frontal and temporal atrophy, thinning of the hypothalamic floor and the CC, and shrinkage of the MBs^12,13^. Particularly, shrinkage of the MBs is a characteristic finding for CTE and is one of the supportive neuropathological findings in the consensus criteria^26^. Furthermore, MB dysfunction is suggested to play a significant role in producing memory loss, cognitive impairment, and eventual dementia^27^. However, the exact mechanism underlying MB shrinkage in CTE is unknown.

One hypothesis for the cause of MB atrophy in rmTBI is a direct traumatic injury to the hypothalamus, including the MBs. The hypothalamus is one of the brain regions vulnerable to injury from head trauma. A report based on 15 cases of severe TBI identified four anatomical lesions by examining serial histological sections of the hypothalamus: (1) lesions of the supraoptic and paraventricular nuclei, (2) lesions of the infundibulum, (3) lesions in and around the third ventricle, and (4) lesions of the MBs. Lesions in all four areas were consistently found in all TBI patients^28^. Furthermore, infarction and hemorrhage were less frequently observed in the MBs, but gliosis and loss of neurons were observed, especially in longstanding cases^28^. In previous MR imaging analyses, detected disruption of fornical fibers after TBI, including the MBs, using diffusion tensor imaging with anisotropy measures and tractography^29–31^. In addition, a reduction in the volume of the fornix and atrophy elsewhere, including the CC and hippocampus, were observed in a long-term course after TBI^32,33^. These neuropathological and neuroimaging results may help explain the cause of the rmTBI-related MB atrophy in our study.

Another potential mechanism for MB atrophy in rmTBI participants is neurodegeneration. Axonal injury and rmTBI might trigger molecular pathways that result in the aggregation of proteins prone to pathological accumulation in neurodegenerative disease, including phosphorylated tau (p-tau), TAR DNA-binding protein 43 kDa, α-synuclein, and amyloid-ß, thereby increasing the likelihood of frontotemporal lobar degeneration, Lewy body disease, or AD^27,29^. Multiple epidemiological studies have shown that trauma is a risk factor for dementia, especially AD^29^. Among neuropathological findings in patients with early-stage AD, only 60% of the patients had senile plaques and/or NF neurofibrillary tangles in the MBs^20^. In addition, age and dementia are accompanied by a loss of MB volume without neuronal loss^30^ or neuronal atrophy^31^. Furthermore, abnormal p-tau immunoreactive neuronal and astrocytic aggregates in subcortical nuclei, including the MBs, are a supportive neuropathological feature in the Preliminary National Institute of Neurological Disorders and Stroke criteria for the pathologic diagnosis of CTE^27^. However, not a single study to date has succeeded in demonstrating that MB atrophy is directly linked to TBI-induced tau accumulation or other TBI-related neurodegenerative changes. Thus, these data suggest that MB atrophy in rmTBI may not only be affected by neurodegenerative disease but may also be produced by direct traumatic injury or by lesions coexisting in other memory-related areas, such as the medial thalamus, mammillothalamic tract, descending columns of the fornix, amygdalofugal pathways, or by other neuronal pathologies.

The current study showed that, in rmTBI participants, MB atrophy occurs, and the MBs volume/TBV ratio correlates with the ROCFT delayed recall. To the best of our knowledge, this is the first study to quantitatively evaluate the relationship between the MBs volume and memory performance in rmTBI participants. Our findings suggest that MB atrophy may serve as an index for the severity of visual memory impairment in rmTBI participants. Furthermore, memory impairment may be attributed to a disconnection between the MBs and the anterior nuclei of the thalamus, which are a part of the Papez circuit. Previous reports on retired National Football League (NFL) players with a history of concussions demonstrated significant reductions in hippocampal volumes and deficits in episodic memory^15^. In addition, after the late 50 s, atrophy of the hippocampus was more pronounced in the players than in HCs^15^. In our study, 85% of the rmTBI participants were aged < 55 years; the hippocampal volume was not significantly different from that of the HC group in the NFL study^15^. It should be noted that our results suggest that the MB atrophy in rmTBI precedes the volume reduction of the hippocampus and may be related to memory impairment in the early stage.

The present study also revealed atrophy of the CC in rmTBI participants, The present study also demonstrated the CC/TBV was smaller in the rmTBI group but not significant after multiple comparisons, as measured by volumetric analysis. The CC is particularly vulnerable to shearing forces or rotation of the brain due to trauma^32^. Our results are consistent with those of previous studies on CC injury in concussion or mild TBI^33^. The CC is crucial for the normal interhemispheric transfer of sensory, motor, attentional, and executive information^34^. Anterior callosal regions, such as the genu, contain fibers from the prefrontal cortex, whereas the body (proceeding from front to back) contains fibers from the premotor, motor, somatosensory, parietal, and temporal cortices^35^. Previous studies suggested that trauma-induced atrophy of the CC likely occurs as a consequence of complex neuropathological features. Another report suggested that active boxers had an average yearly rate of decline in the volumes of the central corpus callosum, but retired boxers did not show progression of atrophy in the CC^36^. These features involve both direct trauma to the CC^37^ and secondary Wallerian degeneration occurring because of diffuse damage elsewhere in the brain, disrupting the integrity of white matter tracts^38^. Other studies have reported an association between white matter alterations (e.g., volume and microstructure) and cognitive function in TBI^39^. Our study showed that the CC volume/TBV ratio was not correlated with age, duration since the first injury, or any cognitive assessment results. This result may reflect the heterogeneity of CC damage and widespread white matter alternation in rmTBI participants.

This study had some limitations. First, the relatively small sample size of this study requires caution in interpreting the results, and the study should be conducted with a larger sample. Second, only Japanese participants were evaluated; thus, the results should be interpreted cautiously because post-TBI outcomes differ according to genetic associations among Caucasians, African-Americans, Hispanic, and Asians^40^. In addition, racial variations in physique may contribute to the extent of brain trauma. Third, the sports professions varied. A previous study found differences in impact velocity and head injury criteria between boxing and American football^41^. Therefore, differences in MB or CC atrophy in rmTBI should be examined by sport. Future research should investigate the longitudinal associations between MB atrophy and the prognosis of behavioral or cognitive impairment in rmTBI to determine whether MB atrophy can be used as an imaging marker. Fourth, previous reports have indicated that mild TBI often remains undiagnosed and untreated^42^, and rmTBI patients with severe post-injury symptoms exhibit stronger recall bias^43^. Therefore, an exact number of rmTBI is difficult to estimate using self-reported information and the number of confirmed diagnoses by a physician. Fifth, the lack of neuropsychological test data from healthy controls makes clinical interpretation of cognitive dysfunction in rmTBI participants difficult. Sixth, although no participants showed abnormal findings on conventional T2WI, FLAIR, or SWI, the possibility that diffuse axonal injury without microbleeds affected the current results couldn’t be ruled out. Finally, there were limitations in controlling for lifestyle, genetic background, and environmental factors of the participants to demonstrate the association between rmTBI and MB atrophy.

In conclusion, retired athletes with a long history of rmTBI have MB atrophy, and this may be related to memory impairment associated with disconnection of the MBs from the Papez circuit.

### Supplementary Information

Supplementary Tables.

## Data Availability

The datasets generated and/or analyzed during the current study are not publicly available due to the anonymity of the patients but are available from the corresponding author on reasonable request.
