# Supplementary material for: Association between mammillary body atrophy and memory impairment in retired athletes with a history of repetitive mild traumatic brain injury
Source: Sci Rep. 2024 Mar 26;14:7129. doi: 10.1038/s41598-024-57383-6 (PMC10965940; doi:10.1038/s41598-024-57383-6)
Supplement: Supplementary file 1 — Supplementary Tables. [file 41598_2024_57383_MOESM1_ESM.docx]

| Table 1. Group differences in brain volumes compared with retired athletes with rmTBI and HC in Reader 2 | | | | | | |
| --- | --- | --- | --- | --- | --- | --- |
|  | Volume, cm³, mean ± SD | |  | Regional volume/TBV ratio, ×10^-3^ mean ± SD | |  |
|  | Retied athletes with rmTBI | HC | p value | Retied athletes with rm TBI | HC | p value |
|  | n = 27 | n = 23 |  | n = 27 | n = 23 |  |
| TBV | 1.12 ± 0.13 ×10³ | 1.17 ± 0.12 ×10³ | ns | ― | ― | ― |
| MB | 0.15 ± 0.06 | 0.23 ± 0.02 | 0.002** | 0.13 ± 0.05 | 0.20 ± 0.03 | <0.001** |
| Put | 10.7 ± 1.46 | 10.9 ± 1.21 | ns | 9.62 ± 1.06 | 9.31 ± 0.89 | ns |
| GP | 3.16 ± 0.40 | 3.12 ± 0.47 | ns | 2.86 ± 0.41 | 2.68 ± 0.41 | ns |
| CN | 8.77 ± 1.43 | 9.30 ± 1.19 | ns | 7.85 ± 0.87 | 7.93 ± 0.76 | ns |
| Thal | 13.9 ± 2.19 | 15.1 ± 1.96 | 0.02* | 12.5 ± 1.65 | 13.6 ± 1.72 | ns |
| Hipp | 7.41 ± 1.04 | 7.72 ± 1.12 | ns | 6.65 ± 0.67 | 6.60 ± 0.78 | ns |
| Amy | 3.96 ± 0.61 | 4.03 ± 0.53 | ns | 3.55 ± 0.42 | 3.45 ± 0.45 | ns |
| CC | 13.9 ± 1.65 | 15.9 ± 2.17 | 0.02* | 12.5 ± 1.65 | 13.6 ± 1.72 | 0.04* |
| Abbreviations: rmTBI; repetitive mild traumatic brain injury, HC; healthy control, SD; standard deviation, TBV; total brain volume, ns; not significant, MB; mammillary bodies, Put; putamina, GP; globus pallida, CN; caudate nuclei, Thal; thalami, Hipp; hippocampi, Amy; amygdalae, CC; corpus callosum | | | | | | |
| ** p < 0.006, * p <0.05 | | | | | | |

| Table 2. Correlation analysis of brain volumes and clinical parameters in retired athletes with rmTBI in Reader 2 | | | | | | |
| --- | --- | --- | --- | --- | --- | --- |
|  |  |  | the MB volume / TBV ratio | | the CC volume / TBV ratio | |
|  |  | Mean ± SD, Range | ρ | p value | ρ | p value |
| Age | | 43.9 ± 10.9, 31-69 | 0.22 | ns | 0.32 | ns |
| Year of education | | 13.7 ± 2.4, 9-18 | 0.02 | ns | 0.10 | ns |
| Years since the first injury | | 26.4 ± 11.6, 9-47 | 0.18 | ns | 0.25 | ns |
| Duration of exposure to rmTBI | | 15.4 ± 9.4, 12-25 | 0.38 | ns | -0.01 | ns |
| Cognitive assessments, Score | |  |  |  |  |  |
| MMSE |  | 26.5 ± 3.1, 15-30 | 0.45 | 0.03* | 0.24 | ns |
| LM | Immediate recall | 8.8 ± 5.0, 0-20 | 0.37 | ns | 0.12 | ns |
|  | Delayed recall | 6.2 ± 5.4, 0-20 | 0.53 | 0.006* | 0.17 | ns |
| ROCF test | Copy condition | 34.8 ± 1.9, 29-36 | 0.11 | ns | 0.03 | ns |
|  | Delayed recall | 16.1 ± 7.3, 4-29 | 0.58 | 0.003** | -0.05 | ns |
| WCST | Categories achieved | 4.1 ± 2.2, 0-7 | 0.29 | ns | -0.12 | ns |
| Stroop | Ⅰ | 16.1 ± 4.0, 10-22 | -1.44 | ns | 0.10 | ns |
|  | Ⅱ | 21.0 ± 7.0, 10-35 | -0.04 | ns | 0.10 | ns |
|  | Ⅲ | 27.5 ± 15.6, 15-87 | -0.10 | ns | -0.02 | ns |
| Abbreviations: rmTBI; repetitive mild traumatic brain injury, MB; mammillary bodies, TBV; total brain volume, CC; corpus callosum, SD; standard deviation, ns; not significant, MMSE; mini-mental state examination, LM; logical memory, ROCF test; Ray-Osterrieth complex figure test, WCST; Wisconsin card sorting test Keio version | | | | | | |
| ** p < 0.005, * p <0.05 | | | | | |  |
